# Crystallization and preliminary X-ray diffraction analysis of the amidase domain of allophanate hydrolase from *Pseudomonas* sp. strain ADP

**DOI:** 10.1107/S2053230X13034705

**Published:** 2014-02-19

**Authors:** Sahil Balotra, Janet Newman, Nigel G. French, Lyndall J. Briggs, Thomas S. Peat, Colin Scott

**Affiliations:** a Ecosystem Sciences, CSIRO, GPO Box 1700, Canberra, ACT 2601, Australia; b Materials, Science and Engineering, CSIRO, 343 Royal Parade, Parkville, VIC 3052, Australia

**Keywords:** AtzF, allphanate hydrolase, amidase domain, *Pseudomonas* sp. strain ADP

## Abstract

The allophanate hydrolase from *Pseudomonas* sp. strain ADP was expressed and purified, and a tryptic digest fragment was subsequently identified, expressed and purified. This 50 kDa construct retained amidase activity and was crystallized. The crystals diffracted to 2.5 Å resolution and adopted space group *P*2_1_, with unit-cell parameters *a* = 82.4, *b* = 179.2, *c* = 112.6 Å, β = 106.6°.

## Abstract 2

The amidase domain of the allophanate hydrolase AtzF from *Pseudomonas* sp. strain ADP has been crystallized and preliminary X-ray diffraction data have been collected.

## 1. Introduction   

Allophanate hydrolase (EC 3.5.1.54) from *Pseudomonas* sp. strain ADP (AtzF) is a member of the amidase family of enzymes that possess a conserved serine- and glycine-rich motif, the so-called ‘amidase signature’ sequence (Shapir *et al.*, 2005 ▶). The amidases are serine hydrolases that catalyze the hydrolysis of an amide group to a carboxylic acid with the concomitant release of ammonia. These enzymes adopt a classic α-β-α fold and possess a Ser-*cis*Ser-Lys catalytic triad that is conserved throughout the family (Shin *et al.*, 2002 ▶). The catalytic serine in AtzF (Ser189) has been identified experimentally (Shapir *et al.*, 2005 ▶). AtzF contains 605 amino acids with an estimated molecular weight of 64 kDa, while the estimated native molecular weight has been shown to be approximately 260 kDa (Shapir *et al.*, 2005 ▶), suggesting that the native functional enzyme is a tetramer. The substrate range of allophanate hydrolase is quite narrow, and to date allophanate, malonamate and biuret are the only known substrates of this enzyme (Shapir *et al.*, 2005 ▶, 2006 ▶). The native substrate of AtzF, allophanate, is generated by two biological processes: cyanuric acid mineralization and urea decomposition (Shapir *et al.*, 2005 ▶; Kanamori *et al.*, 2004 ▶).

**Figure 1:**
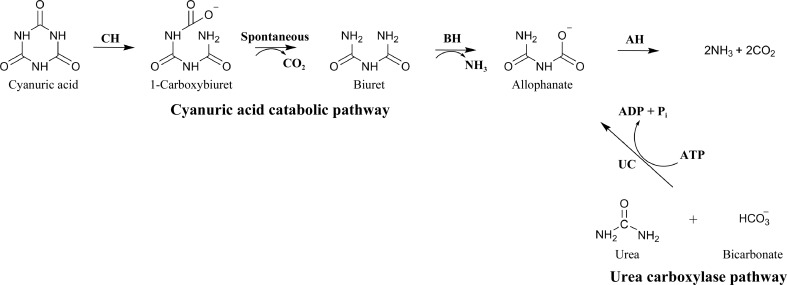
Catabolic processes involving allophanate hydrolase (AH). The cyanuric acid catabolic pathway involves ring opening of cyanuric acid by cyanuric acid hydrolase (CH), spontaneous decarboxylation of the product (1-carboxybiuret) and deamination of biuret by biuret hydrolase (BH) to produce allophanate. The urea catabolic pathway involves ATP-dependent urea carboxylation by urea carboxylase (UC) to produce allophanate. Both pathways then depend upon allophanate deamination by allophanate hydrolase to avoid spontaneous decarboxylation (and urea formation).

In most ureolytic organisms, the nickel-containing metalloenzyme urease (EC 3.5.1.5) provides a single-step hydrolysis of urea that yields ammonia and carbamate (Blakeley *et al.*, 1969 ▶), which spontaneously decays into ammonia and carbon dioxide (Carter *et al.*, 2009 ▶). However, there is an alternate urea metabolism pathway (Fig. 1 ▶), the urea carboxylate pathway, which is used by some algae and fungi (Strope *et al.*, 2011 ▶). In this pathway, urea carboxylase first catalyzes the ATP-dependent carboxylation of urea to form allophanate, which is subsequently deaminated by allophanate hydrolase, leading to the production of ammonia and carbon dioxide (Kanamori *et al.*, 2004 ▶; Maitz *et al.*, 1982 ▶; Roon & Levenberg, 1972 ▶).

Allophanate hydrolase also participates in the cyanuric acid mineralization pathway (Fig. 1 ▶), in which the cyanuric acid ring is hydrolytically opened by cyanuric acid hydrolase (AtzD or TrzD; EC 3.5.2.15) forming the unstable metabolite carboxybiuret, which spontaneously decarboxylates to form biuret (Peat *et al.*, 2013 ▶; Udiković-Kolić *et al.*, 2012 ▶). Allophanate is produced from biuret by AtzE (biuret hydrolase; EC 3.5.1.84) *via* a single deamination (Martinez *et al.*, 2001 ▶). Hydrolysis of allophanate is then carried out by allophanate hydrolase (Martinez *et al.*, 2001 ▶).

Here, we report the expression, purification, crystallization and initial X-ray diffraction analysis of the allophanate hydrolase (AtzF) from *Pseudomonas* sp. strain ADP.

## 2. Materials and methods   

### 2.1. Cloning   

The *atzF* gene was obtained from GenScript (New Jersey, USA); the coding sequence used was identical to that described by Martinez *et al.* (2001 ▶) (accession No. U66917) and was provided as an *Nde*I/*Bam*HI insert in pUC57 (supplied by GenScript; Table 1 ▶). The *Nde*I/*Bam*HI restriction fragment containing the *atzF* gene was subcloned into the *Nde*I and *Bam*HI sites of pETCC2 (Peat *et al.*, 2013 ▶).

For the construction of the gene encoding C-terminally truncated AtzF (AtzF_467_), primers were designed to amplify the region corresponding to the first 1404 bp of the *atzF* gene (AtzF Rev Trunc; Table 1 ▶). Phusion DNA polymerase and dNTPs were purchased from New England Biolabs (Ipswich, USA). AtzF Fwd A and AtzF Rev Trunc primers (Table 1 ▶) were used to amplify the targeted region of *atzF*. The reaction conditions for PCR were 1× Phusion HF Buffer, 2 pg pETCC2-*atzF* DNA template, 0.5 µ*M* primers, 200 µ*M* dNTPs and one unit of Phusion DNA polymerase in a total reaction volume of 50 µl. The cycle conditions for the PCR reaction were a 30 s denaturation step at 98°C followed by annealing at 53°C for 20 s and then extension for 120 s at 72°C for 30 cycles. The amplicon was separated on 0.6% agarose gel by electrophoresis to confirm the size of the amplicon. The DNA band was excised from the gel and was purified using a NucleoSpin Gel and a PCR Clean-up kit (Macherey-Nagel). The purified DNA was double digested with *Bam*HI-HF and *Nde*I enzymes (NEB) before it was cloned into pETCC2 vector at *Bam*HI and *Nde*I restriction sites using T4 DNA ligase (NEB) as per the manufacturer’s instructions.

### 2.2. Expression and purification of AtzF and AtzF_467_   

pETCC2 derivatives containing either *atzF* or *atzF*_467_ were used to transform electrocompetent *Escherichia coli* BL21 (λDE3) cells (Invitrogen) and were grown at 310 K on Luria–Bertani (LB), Miller (Miller, 1972 ▶) agar plates supplemented with 100 µg ml^−1^ ampicillin. Overnight starter cultures of 50 ml were inoculated with 4–5 colonies. The overnight cultures were diluted 1:20 into 950 ml LB, Miller medium and shaken at 310 K and 200 rev min^−1^ until an OD_600_ of 0.6–0.8 was attained. Protein expression was initiated by the addition of 100 µ*M* isopropyl β-d-1-thiogalactopyranoside (IPTG). The induced cultures were kept at 310 K overnight whilst shaking at 200 rev min^−1^.

The cells were harvested by centrifugation at 4000*g* for 10 min in an Avanti J-E centrifuge (Beckman Coulter, Indianapolis, USA) and then resuspended in lysis buffer (50 m*M* HEPES, 100 m*M* NaCl pH 7.5 for AtzF and 50 m*M* Tris, 100 m*M* NaCl pH 7.5 for AtzF_467_) and lysed by passage through an Avestin C3 homogenizer three times at 124 MPa. Insoluble cellular debris was removed by centrifugation at 21 000*g* using an Avanti J-E centrifuge.

Wild-type AtzF was purified from the soluble cell-free extract in three steps: His-tag affinity chromatography using an Ni–NTA Superflow cartridge (Qiagen, Maryland, USA) with a gradient of 0–­300 m*M* imidazole in 50 m*M* HEPES, 100 m*M* NaCl pH 7.5, anion exchange using a HiTrap Q HP column (GE Healthcare Life Sciences, Uppsala, Sweden) and a gradient of 100–1000 m*M* NaCl in 50 m*M* HEPES pH 7.5, and finally size-exclusion chromatography using a 130 ml column packed with Superdex 200 prep-grade resin (GE Healthcare Life Sciences) with a buffer comprised of 50 m*M* HEPES, 100 m*M* NaCl pH 7.5. 20 column volumes of buffer were used to achieve the gradients used for the Ni–NTA Superflow cartridge and HiTrap Q HP purification steps. The protein was concentrated in an Amicon Ultra-15 Centrifugal Filter Unit with an Ultracel-30 membrane (Millipore, Carrigtwohill, Ireland) to 5 mg ml^−1^ and snap-frozen in liquid nitrogen in 100 ml aliquots. The final purity was estimated to be 98% from a Coomassie-stained gel, and typical yields were 18–20 mg purified protein from 1 l LB medium.

AtzF_467_ was purified from soluble cell-free extract in two steps: binding to an Ni–NTA column followed by a wash step with 50 m*M* Tris, 100 m*M* NaCl pH 7.5 buffer and then by two wash steps with 10 m*M* imidazole and 20 m*M* imidazole in 50 m*M* Tris, 100 m*M* NaCl pH 7.5 buffer. Finally, the protein was eluted with 250 m*M* imidazole. The protein was further purified by size-exclusion chromatography using the same column as used for AtzF but in 50 m*M* Tris, 100 m*M* NaCl pH 7.5 buffer. The final purity was estimated to be 98% from a Coomassie-stained gel, and typical yields were 70–75 mg purified protein from 1 l LB medium. All protein chromatographic work was conducted using an ÄKTApurifier UPC 10 (GE Healthcare Life Sciences). The purified protein was concentrated to about 5 mg ml^−1^ and snap-frozen (using liquid nitrogen) in 50–100 µl aliquots.

Both purified AtzF and AtzF_467_ include 20-amino-acid N-terminal extensions that include a thrombin cleavage site and a hexahistidine tag (Table 1 ▶).

### 2.3. Proteolysis and N-terminal sequencing   

**Figure 2:**
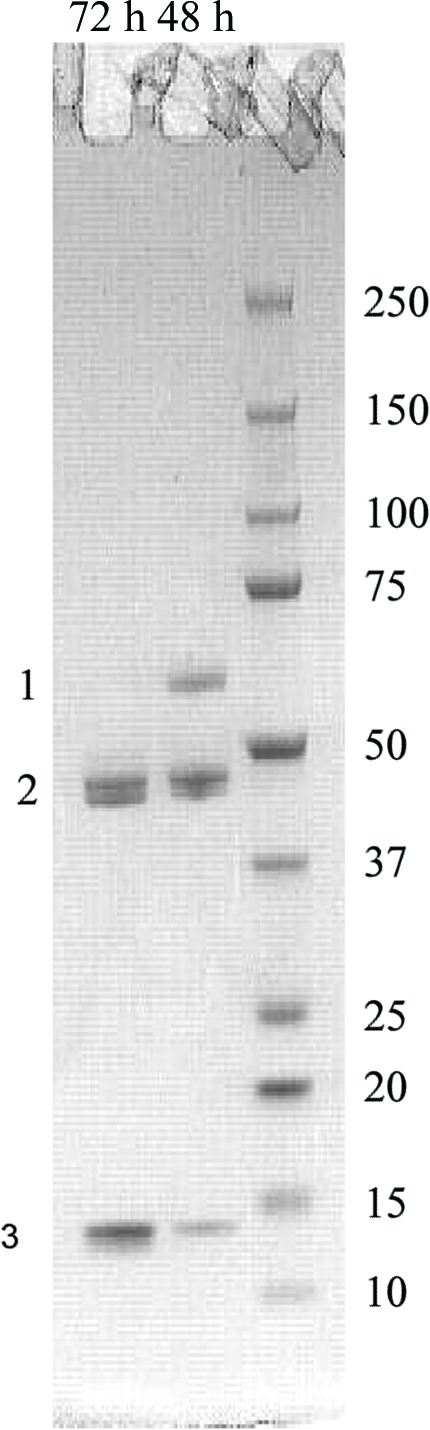
Fragmentation patterns from AtzF trypsinolysis. Tryptic digestion of AtzF resulted in the fragmentation of AtzF into two distinct bands on SDS–PAGE. The lane next to the ladder (right lane; labelled in kDa) shows a partial digest of AtzF 48 h after adding trypsin. The highest molecular weight band (labelled 1) represents undigested AtzF. The two bands adjacent to each other at nearly 48 kDa (labelled 2) possessed the same peptide sequence (as determined by N-terminal sequencing) and contain the N-terminus. The smallest visible band (labelled 3) is the 14 kDa C-­terminal fragment of AtzF.

Limited proteolysis of full-length AtzF (0.8 mg ml^−1^) was carried out with 0.04 mg ml^−1^ trypsin (MP Biomedicals, Australia) at 293 K for 48 h. Tryptic fragments were separated by SDS–PAGE (Shapiro *et al.*, 1967 ▶) using Tris–HEPES 10–20% precast gels (NuSep; Fig. 2 ▶). N-­terminal sequencing of the proteolytic products was used to identify the position of the proteolysis site (Australian Proteome Analysis Facility, Sydney, Australia).

### 2.4. Activity test for full-length AtzF and AtzF_467_   

Biuret (Sigma–Aldrich, St Louis, USA) was used as a substrate to determine the activity of the wild-type and truncated enzymes. The hydrolytic production of ammonia from biuret by AtzF and truncated AtzF was measured using an ammonia assay kit (Sigma–Aldrich). Briefly, l-glutamate dehydrogenase (GDH) reductively aminates α-­ketoglutaric acid in an NADPH-dependent reaction (Bergmeyer, 1990 ▶). The consumption of NADPH results in a decrease in the UV absorbance at 340 nm. A SpectraMax M2 spectrophotometer (Molecular Devices, California, USA) was used to follow the decrease in absorbance in this assay. The assay was performed in 280 m*M* TAPS buffer pH 9; the concentrations and components of the reaction were 10 µg BSA, 0.5 m*M* NADPH, 10 m*M* α-keto­glutaric acid, 50 m*M* biuret, 191 pmol AtzF or AtzF_467_, 0.875 U GDH.

An ammonia standard (150 µ*M*) provided with the kit was used as a positive control. Enzyme-free negative controls contained 50 m*M* biuret in addition to the kit reagents and a substrate-free control contained 191 pmol AtzF or AtzF_467_ along with the kit reagents.

### 2.5. Stability analysis   

**Figure 3:**
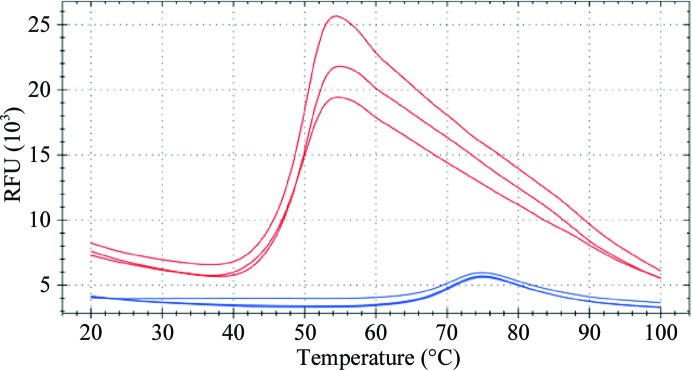
Melting curve. A temperature melting curve of AtzF was performed in triplicate, using differential scanning fluorimetry with the dye SYPRO Orange. The red curves are the AtzF protein (in triplicate) in the buffer used for crystallization (50 m*M* HEPES, 100 m*M* NaCl pH 7.5). The blue curves are a 0.1 mg ml^−1^ lysozyme control.

Heat-denaturation curves of the full-length AtzF protein, along with AtzF_467_, were generated using differential scanning fluorimetry. The two samples were tested in a suite of different buffers and pHs in triplicate (‘buffer screen 9’; Seabrook & Newman, 2013 ▶). The assay was performed in a CFX96 RT-PCR machine (Bio-Rad) with 19.6 µl of each screening condition, 300 nl protein at 5–6 mg ml^−1^ and 300 nl of a 1:10 (aqueous) dilution of SYPRO Orange dye (Sigma). Both proteins displayed curves indicative of a well folded protein: AtzF in the buffer used in the final chromatography step (50 m*M* HEPES, 100 m*M* NaCl pH 7.5) displayed a *T*_m_ of 50.4°C and AtzF_467_ in 50 m*M* Tris pH 7.5, 100 m*M* NaCl displayed a melting temperature 2°C higher (Fig. 3 ▶)

### 2.6. Crystallization   

**Figure 4:**
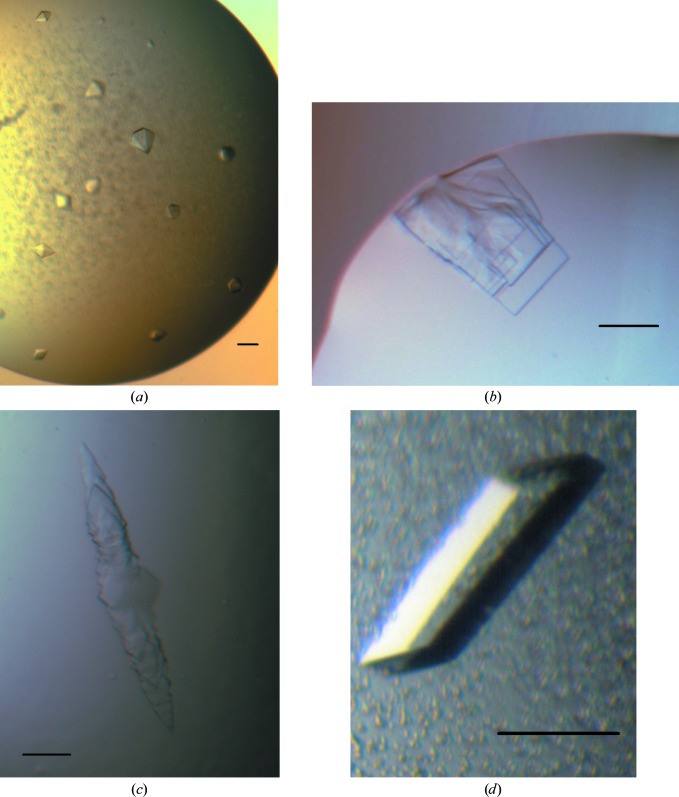
AtzF crystals. The scale bar represents 100 µm in each case. (*a*) Bipyramidal crystals of AtzF produced in 4 *M* sodium formate. These crystals diffracted X-rays to about 6.5 Å resolution and could not be reproduced. (*b*) Intergrown plates of trypsin-treated AtzF (*in situ* proteolysis) grown from 1 *M* ammonium sulfate, 1 *M* lithium sulfate, 0.1 *M* Tris chloride pH 8.5. (*c*) Intergrown plates of AtzF467 grown from 20%(*w*/*v*) PEG 6000, 0.1 *M* Na MES pH 6.5, 0.2 *M* calcium chloride. (*d*) Truncated AtzF crystals grown from 11%(*w*/*v*) PEG 3350, 2% Tacsimate pH 5 microseeded with the crystals shown in (*c*). These crystals diffracted X-rays to 2.5 Å resolution and were used for data collection.

All crystallization experiments were performed in 96-well SD-2 plates (IDEX, USA) against a 50 µl reservoir. Most often, the droplets consisted of 150 nl concentrated protein combined with 150 nl reservoir solution. Either a Phoenix (Art Robbins Instruments, USA) or a Mosquito (TTP LabTech, UK) robot was used to place the crystallization drops. Initial screening experiments with two commercial screens (JCSG+ and PACT; Newman *et al.*, 2005 ▶) and a 96-condition pH/salt gradient (PS gradient) screen at two temperatures, 20 and 8°C, showed no hits. The screening was extended to an 8 × 96 condition (768-condition) in-house screen (C3 screen; details of this and the PS gradient screen are available at http://c6.csiro.au), set up both in a conventional manner (droplets against a reservoir of crystallant solution) and against a reservoir of 1.5 *M* NaCl at 20°C. After 35 d, well shaped hexagonal crystals were observed in a drop equilibrated against 4 *M* sodium formate (Fig. 4 ▶). These crystals showed poor diffraction (6.5 Å maximum resolution) on the MX2 (microfocus) beamline of the Australian Synchrotron. These crystals could not be reproduced, even with seeding. *In situ* proteolysis screening experiments were set up using either trypsin or chymotrypsin (1:1000 ratio of protease:AtzF) in the JCSG+, PACT and PS gradient screens at 20°C, and after a month some indications of crystallinity were observed in several ammonium sulfate-containing conditions of the PS gradient screen. The C3_6 screen, which focuses on ammonium sufate-containing conditions, was set up at both 8 and 20°C with trypsin or chymotrypsin-treated protein, and an intergrown plate-shaped crystal was observed with the trypsin-treated protein in a condition consisting of 1 *M* ammonium sulfate, 1 *M* lithium sulfate, 0.1 *M* Tris chloride pH 8.5. Trypsin digests of the full-length protein, followed by mass-spectrometric analysis, revealed one major digestion site, resulting in a large N-terminal domain and a smaller C-­terminal domain of molecular weights 48 and 14 kDa, respectively. Enzyme assays showed that the larger domain retained the amidase activity. An N-terminal His-tagged construct of the larger domain was produced, concentrated to 5.5 mg ml^−1^ and set up against the JCSG+, PACT and PS gradient screens as above, with droplets consisting of 200 nl protein and 200 nl crystallant. The protein crystallized as intergrown plates from PACT condition B9 [0.2 *M* lithium chloride, 0.1 *M* MES pH 6.5, 20%(*w*/*v*) PEG 6K]. Crystals from this droplet were crushed and used to microseed further screening experiments, using in particular PEG-based screens, including PEG/Ion HT from Hampton Research. With seeding, the protein crystallized readily, producing a number of hits in PEG conditions. Crystals with a more robust morphology were found after two weeks in droplets from the PEG/Ion screen containing Tacsimate (Tacsimate is a mixture of organic acids produced by Hampton Research, which comprises 1.8305 *M* malonic acid, 0.25 *M* triammonium citrate, 0.12 *M* succinic acid, 0.3 *M* dl-malic acid, 0.4 *M* sodium acetate, 0.5 *M* sodium formate and 0.16 *M* diammonium tartrate) at pH 5 or lower and 12–16%(*w*/*v*) PEG 3350. Optimization around these conditions and microseeding with the improved crystals led to the production of optimized crystals in conditions consisting of 11–14%(*w*/*v*) PEG 3350, 2% Tacsimate pH 5 at 293 K (Fig. 4 ▶).

### 2.7. X-ray diffraction data collection   

Optimized crystals were tested in a number of cryoprotectants on the MX2 beamline of the Australian Synchrotron. A crystal grown against a reservoir consisting of 11%(*w*/*v*) PEG 3350, 2% Tacsimate pH 5 was briefly introduced to a cryoprotectant consisting of 10% ethylene glycol, 10% glycerol, 80% reservoir solution and then flash-cooled in a stream of cold N_2_ at the beamline. 360° of data (2 s exposure, 1° oscillation, wavelength of 0.9529 Å) were collected.

Data were indexed with *XDS* (Kabsch, 2010 ▶) and scaled using *SCALA* (Evans, 2006 ▶). Molecular replacement using *Phaser* (McCoy *et al.*, 2007 ▶) with PDB entry 2dqn (Nakamura *et al.*, 2006 ▶) as a search model revealed a clear solution with a log-likelihood gain of 757.

## 3. Results and discussion   

AtzF from *Pseudomonas* sp. strain ADP was expressed heterologously in *E. coli* and purified to apparent homogeneity. The yield of purified AtzF varied from 18 to 20 mg per litre of culture. The specific activity of the purified enzyme was determined to be 15.4 µmol min^−1^ pmol^−1^ using 50 m*M* biuret as a substrate. Size-exclusion chromatography (Fig. 2 ▶) suggested that native AtzF was a homohexamer (∼360 kDa), rather than a tetramer (∼240 kDa) as suggested elsewhere (Shapir *et al.*, 2005 ▶).

Thermal stability studies of AtzF showed that the transition from the native to the unfolded state follows a first-order phase transition with a *T*_m_ of 48.3°C (Fig. 3 ▶); however, the protein was recalcitrant to crystallization (Fig. 4 ▶). *In situ* proteolysis crystallization trials resulted in the formation of needle-shaped crystallites (Fig. 4 ▶), which suggested that a fragment of the full-length enzyme produced by a tryptic digest may be more amenable to structural studies.

Scaled-up tryptic proteolysis of the full-length protein produced two major fragments of approximately 48 and 14 kDa (Fig. 2 ▶). N-­terminal sequencing of the smaller 14 kDa band identified the location of the tryptic digestion as Lys467, whereas for the 48 kDa band the location of the tryptic cut was arginine at position 13. This suggested that larger 48 kDa fragment was the N-terminal fragment and the 14 kDa fragment was the C-terminal fragment. Moreover, the 48 kDa fragment was predicted by *pBLAST* to contain the entire amidase domain (*i.e.* the domain that is likely to be responsible for allophanate deamination).

A gene encoding the first 467 amino acids of AtzF (N-terminally histidine-tagged) was expressed and the product was purified to apparent homogeneity. The C-terminally truncated AtzF (AtzF_467_) yielded 3.5–3.8 times more purified protein per litre of medium compared with full-length AtzF (*i.e.* 72–75 mg AtzF_467_ per litre). The specific activity of AtzF_467_ for biuret was 17.6 µmol min^−1^ pmol^−1^ (using 50 m*M* biuret), demonstrating that it was as active as the full-length protein. However, unlike full-length AtzF, AtzF_467_ was shown to be dimeric by size-exclusion chromatography (∼110 kDa; data not shown), suggesting that the C-terminal 14 kDa fragment may play a role in organizing the oligomeric state of AtzF. Thermal melting analysis showed that AtzF_467_ had a slightly higher *T*_m_ (50°C) than AtzF (Fig. 3 ▶).

The AtzF_467_ construct produced large single crystals that were suitable for X-­ray analysis in the presence of Tacsimate at pH 5 when seeded crystals were obtained from the Tascimate-containing PEG/Ion screen. The crystals were of variable quality, despite looking similar, and tens of crystals were tested before finding one [grown in 11%(*w*/*v*) PEG 3350, 2% Tacsimate pH 5] that diffracted isotropically to 2.5 Å resolution, which was used for data collection (Fig. 4 ▶). The crystals adopted space group *P*2_1_, with unit-cell parameters *a* = 82.4, *b* = 179.2, *c* = 112.6 Å, β = 106.6°. In total, 86 96-well crystallization plates were set up to obtain the final well diffracting crystal.

While this manuscript was in preparation, the crystal structure of a full-length allophante hydrolase from the ureolytic yeast *Kluyvero­myces lactis* was published (Fan *et al.*, 2013 ▶). A comparison will be made between the published structure and that of AtzF in the near future.

## Figures and Tables

**Table 1:** DNA and protein used in this study The nucleotide and peptide sequences for the artificial His tag used for the purification of AtzF and AtzF_467_ are shown in bold.

Source organism	*Pseudomonas* sp. strain ADP
DNA source	GenScript, based on accession No. U66917
Gene sequence encoding His-tagged full-length AtzF	**atgggcagcagccatcatcatcatcatcacagcagcggcctggtgccgcgcggcagccat**atgaatgaccgcgcgccccaccctgaaagatctggtcgagtcacgccggatcacctgaccgatctggcttcctatcaggctgcctatgccgccggtacagacgccgccgacgtcatttcggacctgtatgcccgtatcaaagaagacggcgaaaatccgatctggattagcctgttgcccttggaaagcgcattggcgatgctggccgacgcgcagcaacgcaaggacaagggagaagcgttgccgctctttggcatccccttcggcgtcaaggacaacatcgacgtcgcaggccttccgacgactgccgggtgtacggggttcgcgcgtacgccccgacagcacgccttcgtcgtacagcgcctggtggacgctggcgcgatcccgatcggaaaaacgaacctcgatcaattcgcgaccgggttgaacggcactcgcacgccgtttggcattccgcgctgcgtgttcaacgagaactacgtatccggcggctccagcagtggctccgcagtggccgtcgccaacggcacggtaccgttctcgctcgggacggacactgccggttccggccgcattcctgctgcgttcaacaatctggtgggcttgaaaccgaccaaaggcctgttctcgggcagtggactggttcccgcggcgcgaagccttgactgcatcagcgtcctcgcccataccgtagatgacgcccttgcggtcgcacgcgtcgccgccggctacgatgctgatgacgctttttcgcgcaaggcgggcgccgccgcactgacagaaaagagttggcctcgtcgcttcaatttcggggtcccagcggcggaacatcgccagtttttcggtgacgcggaagccgaggcgcttttcaataaagcggttcgcaagcttgaagagatgggtggcacctgcatctcgtttgactatacccccttcaggcaggctgctgaactgctctacgccggcccttgggttgcggagcgcctggcggccatcgagagccttgcggacgagcatcccgaggtgctccacccggtcgttcgtgacatcatcttgtccgcgaagcgaatgagcgcagtcgacacgttcaacggtatctatcgcctggccgaccttgtcagggctgcagagagcacttgggaaaagatcgatgtgatgctgctgccgacggcgccgaccatctacactgtagaagacatgctcgccgatccggtacgcctcaacagcaatctgggcttctacacgaacttcgtgaacttgatggatttgtccgcgattgctgttcccgcaggcttccgaaccaatggcctgccatttggcgtcactttcatcggtcgggcgttcgaagatggggcgatcgcaagcttgggaaaagctttcgtggagcacgacctcgccaagggcaacgcggccacggcggcgccacccaaggataccgtcgcaatcgccgtggtaggtgcacatctctccgaccagcccttgaatcatcagctcacggagagcggcggaaagctacgggcaacaacgcgtactgcgccgggatatgccttgtacgcactccgtgatgcgacgccggctaagcctggaatgttgcgcgaccagaatgcggtcgggagcatcgaagtggaaatctgggatctgccggtcgccgggttcggtgcgtttgtaagtgaaattccggcgccgttgggtatcgggacaataacactcgaagacggcagccatgtgaaaggctttctgtgcgagccacatgccatcgagacggcgctcgacatcactcactacggcggctggcgagcatacctcgcggctcaatag
Gene sequence encoding His-tagged AtzF_467_	**atgggcagcagccatcatcatcatcatcacagcagcggcctggtgccgcgcggcagccat**atgaatgaccgcgcgccccaccctgaaagatctggtcgagtcacgccggatcacctgaccgatctggcttcctatcaggctgcctatgccgccggtacagacgccgccgacgtcatttcggacctgtatgcccgtatcaaagaagacggcgaaaatccgatctggattagcctgttgcccttggaaagcgcattggcgatgctggccgacgcgcagcaacgcaaggacaagggagaagcgttgccgctctttggcatccccttcggcgtcaaggacaacatcgacgtcgcaggccttccgacgactgccgggtgtacggggttcgcgcgtacgccccgacagcacgccttcgtcgtacagcgcctggtggacgctggcgcgatcccgatcggaaaaacgaacctcgatcaattcgcgaccgggttgaacggcactcgcacgccgtttggcattccgcgctgcgtgttcaacgagaactacgtatccggcggctccagcagtggctccgcagtggccgtcgccaacggcacggtaccgttctcgctcgggacggacactgccggttccggccgcattcctgctgcgttcaacaatctggtgggcttgaaaccgaccaaaggcctgttctcgggcagtggactggttcccgcggcgcgaagccttgactgcatcagcgtcctcgcccataccgtagatgacgcccttgcggtcgcacgcgtcgccgccggctacgatgctgatgacgctttttcgcgcaaggcgggcgccgccgcactgacagaaaagagttggcctcgtcgcttcaatttcggggtcccagcggcggaacatcgccagtttttcggtgacgcggaagccgaggcgcttttcaataaagcggttcgcaagcttgaagagatgggtggcacctgcatctcgtttgactatacccccttcaggcaggctgctgaactgctctacgccggcccttgggttgcggagcgcctggcggccatcgagagccttgcggacgagcatcccgaggtgctccacccggtcgttcgtgacatcatcttgtccgcgaagcgaatgagcgcagtcgacacgttcaacggtatctatcgcctggccgaccttgtcagggctgcagagagcacttgggaaaagatcgatgtgatgctgctgccgacggcgccgaccatctacactgtagaagacatgctcgccgatccggtacgcctcaacagcaatctgggcttctacacgaacttcgtgaacttgatggatttgtccgcgattgctgttcccgcaggcttccgaaccaatggcctgccatttggcgtcactttcatcggtcgggcgttcgaagatggggcgatcgcaagcttgggaaaagctttcgtggagcacgacctcgccaag
Forward primer (AtzF Fwd A)	ATCATCACAGCAGCGGCCTG
Reverse primer (AtzF Rev Trunc)	GTTTGGTTGGATCCTCATTACTTGGCGAGGTCGTGCTCCACGAAAGC
Cloning vector	pUC57
Expression vector	pETCC2, a pET-14b derivative (Peat *et al.*, 2013 ▶)
Expression host	*E. coli* BL21 (λDE3), Invitrogen
Complete amino-acid sequence of AtzF	**MGSSHHHHHHSSGLVPRGSH**mndraphpersgrvtpdhltdlasyqaayaagtdaadvisdlyarikedgenpiwisllplesalamladaqqrkdkgealplfgipfgvkdnidvaglpttagctgfartprqhafvvqrlvdagaipigktnldqfatglngtrtpfgiprcvfnenyvsggsssgsavavangtvpfslgtdtagsgripaafnnlvglkptkglfsgsglvpaarsldcisvlahtvddalavarvaagydaddafsrkagaaaltekswprrfnfgvpaaehrqffgdaeaealfnkavrkleemggtcisfdytpfrqaaellyagpwvaerlaaiesladehpevlhpvvrdiilsakrmsavdtfngiyrladlvraaestwekidvmllptaptiytvedmladpvrlnsnlgfytnfvnlmdlsaiavpagfrtnglpfgvtfigrafedgaiaslgkafvehdlakgnaataappkdtvaiavvgahlsdqplnhqltesggklrattrtapgyalyalrdatpakpgmlrdqnavgsieveiwdlpvagfgafvsepaplgigtitledgshvkgflcephaietaldithyggwraylaaq
Complete amino-acid sequence of AtzF_467_	**MGSSHHHHHHSSGLVPRGSH**mndraphpersgrvtpdhltdlasyqaayaagtdaadvisdlyarikedgenpiwisllplesalamladaqqrkdkgealplfgipfgvkdnidvaglpttagctgfartprqhafvvqrlvdagaipigktnldqfatglngtrtpfgiprcvfnenyvsggsssgsavavangtvpfslgtdtagsgripaafnnlvglkptkglfsgsglvpaarsldcisvlahtvddalavarvaagydaddafsrkagaaaltekswprrfnfgvpaaehrqffgdaeaealfnkavrkleemggtcisfdytpfrqaaellyagpwvaerlaaiesladehpevlhpvvrdiilsakrmsavdtfngiyrladlvraaestwekidvmllptaptiytvedmladpvrlnsnlgfytnfvnlmdlsaiavpagfrtnglpfgvtfigrafedgaiaslgkafvehdlak
